# ‘Sink or Swim’: A Qualitative Study to Understand How and Why Nurses Adapt to Support the Implementation of Integrated Diabetes Care

**DOI:** 10.5334/ijic.4215

**Published:** 2019-04-03

**Authors:** Fiona Riordan, Niamh McGrath, Sean F. Dinneen, Patricia M. Kearney, Sheena M. McHugh

**Affiliations:** 1 School of Public Health, Western Gateway Building, University College Cork, IE; 2 Centre for Diabetes, Endocrinology and Metabolism, Galway University Hospitals, School of Medicine, National University of Ireland, Galway, IE

**Keywords:** Clinical Nurse Specialists, integrated care, quality improvement, diabetes mellitus

## Abstract

**Background:**

Integrated care, organising care delivery within and between services, is an approach to improve the quality of care. Existing specialist roles have evolved to work across settings and services to integrate care. However, there is limited insight into how these expanded roles are implemented, including how they may be shaped by context. This paper examines how new diabetes nurse specialists working across care boundaries, together with hospital-based diabetes nurse specialists, adapt to support the implementation of integrated care.

**Methods:**

We conducted semi-structured focus groups and interviews with diabetes nurse specialists purposively sampled by work setting and health service region (n = 30). Analysis was data-driven, coding actions or processes to stay closer to the data and using *In Vivo* codes to preserve meaning.

**Findings:**

Community nurse specialists described facing a choice of “sink or swim” when appointed with limited guidance on their role. To ‘swim’ and implement their role, required them to use their initiative and adapt to the local context. When first appointed, both community and hospital nurse specialists actively managed misconceptions of their role by other staff. To establish clinics in general practices, community nurse specialists capitalised on professional contacts to access GPs who might utilise their role. They built GP trust by adopting practice norms and responding to individual needs. They adapted to the lack of a multidisciplinary team “safety net” in the community, by “practicing at a higher level”, working more autonomously. Developing professional links and pursuing on-going education was a way to create an alternative ‘safety net’ so as to feel confident in their clinical decision-making when working in the community. Workarounds facilitated information flow (i.e. patient blood results, treatment, and appointments) between settings in the absence of an electronic record shared between general practices and hospital settings.

**Conclusions:**

Flexibility and innovation facilitates a new way of working across boundaries. Successful implementation of nurse specialist-led integrated care requires strategies to address elements in the inner (differences in practice organisation, role acceptance) and outer (information systems) context.

## Introduction

Integrated care is seen as a way to improve both the quality and efficiency of healthcare delivery for people with chronic conditions [1]. Models of integrated chronic disease management often focus on ‘vertical’ integration between different services and settings, for example community-based and specialist services [2,3,4]. Intermediary support provided by community-based multidisciplinary teams [2,5,6], or the expansion of nurse specialist roles in the community to provide support for primary care [2,3,4,7,8] are strategies that have been adopted to integrate diabetes care in Ireland and internationally. These models deliver better clinical outcomes for patients [2,6,7,9], reduce referrals to secondary care [8], and prevent hospitalisations [5]. However, models of integrated care do not always deliver improvements [10,11,12]. Implementing them successfully within different healthcare or policy contexts can be challenging; health care systems are inherently complex, characterised by unpredictability and self-organising practices [13], making it difficult to introduce and embed change. Moreover, health systems are traditionally designed for delivery of acute or episodic care and not necessarily configured for integrated care.

Interventions are often adapted during implementation to increase compatibility and ‘fit’ with the given context [14]. International research highlights the importance of context in the implementation and success of integrated care [15,16,17,18]. Integration can be supported by existing relationships and shared values between organisations and individuals[15] and a culture of interdisciplinary work [15,17]. Professional networks can serve as a platform for engagement in service development [16]. Lastly, integration can be supported by financing models which remove competition, placing emphasis on collective rather than individual performance [16]. In contrast, integration has been hindered by an organisational culture of ‘silo-working’ [15,17], difficulties with data-sharing and communication caused by different or unlinked IT systems across settings [15,18], and the failure to secure information-sharing agreements between services [15].

The structure of the health system in Ireland is not necessarily suitable for integrated diabetes care. Primary and secondary care services are funded and resourced separately, chronic disease management is often not well integrated between hospitals and general practice [19], and there is variation in the provision of diabetes management in primary care [20,21]. Efforts to integrate care include a model of integrated care developed by the National Clinical Programme for Diabetes (2010) to improve the quality of care and ensure patients receive care in the most appropriate setting according to the complexity of their condition [22]. To support the delivery of this new model, community-based ‘integrated’ diabetes nurse specialists, who work across primary-secondary care boundaries, were recruited from 2013 onwards to complement the predominantly hospital-based diabetes nurse specialist service. Since 2013 there has been on-going commitment to expanding the advanced nursing infrastructure in Ireland, and a plan for the phased roll-out of community nursing pilot initiatives [23].

Nurse specialists play a central role in the integration of chronic disease management [2,3,4,8], running nurse-led clinics [24,25,26], providing specialist education and support to other professionals [4,7,8,25,26], and liaising with other care providers from multiple specialities and co-ordination of patient care [7,25,26,27]. ‘Integrated’ diabetes nurse specialists reflect an international shift towards expanding nurse specialist support in the community [7,8,24,25]. As a new way of working to support care in a system designed for episodic care, it is important to understand how context shapes the delivery of the role. However, few studies have explored the nurse specialist role as it pertains to the delivery of integrated care in practice [17], including the process by which these models may be adapted during implementation [28]. Our aim therefore was “to understand how diabetes nurse specialists support the implementation of integrated care in a complex health system, including determinants of their behaviours.”

## Methods

### Setting

In Ireland, both hospital and community diabetes nurse specialists support integrated care by managing complex patients with type 2 diabetes, liaising with other professionals, delivering professional and patient education, and nurse-led clinics [25]. While hospital diabetes nurse specialists spent 100% of their Whole Time Equivalent in hospital, new community diabetes nurse specialists are distinct in that they split their Whole Time Equivalent between the community (80%) and hospital (20%) to facilitate integration between the two settings [22].

**Figure 1:**
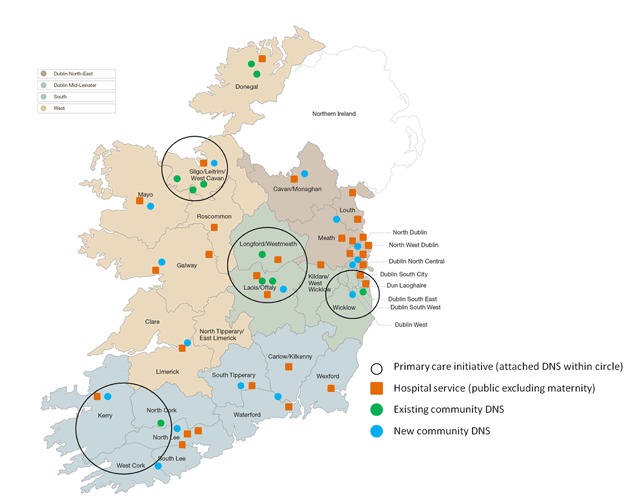
Hospital services, new and existing ‘integrated’ community posts (n = 26) across the four administrative regions of the health service.

At the end of 2016 when this study was carried out, there were 26 nurses in post. Community diabetes nurse specialists include: 1) existing (prior to 2013) community diabetes nurse specialists in areas with local diabetes programmes based in primary care, involving interested professionals aiming to improve the quality of diabetes care at a local level; 2) additional new posts placed into areas with an existing community diabetes nurse specialists; and 3) community diabetes nurse specialists posts entirely new to an area (no previous community diabetes nurse specialists) (Figure 1). At the time of the study, all community diabetes nurse specialists were attached to a hospital and they reported to the Director of Nursing in that hospital.

Programmes: Diabetes in General Practice (69 practices in Cork and Kerry); Health Service Executive (HSE) Midland Diabetes Structured Care Programme (30 practices in Laois, Offaly, Longford and Westmeath); HSE West (19 practices in Sligo and Leitrim); East Coast Area Diabetes Shared Care (25 practices in Dublin South and Wicklow).

### Participants and sampling

Participants were sampled from respondents to a national diabetes nurse specialists survey who indicated their willingness to be contacted about the follow-up qualitative study [25]. Participants were purposively sampled according to their main work setting (hospital or community) across the four administrative regions of the Health Service Executive, the national health system in Ireland (Table 1). A greater proportion of community-based diabetes nurse specialists were sampled to enable detailed exploration of the new integrated care role. Response rate to the initial survey was 67% (n = 101); most of whom (n = 96, 95%) indicated their willingness to be contacted about the follow-up qualitative study [25]. Of 40 diabetes nurse specialists invited, 30 took part in total, in two focus groups (n = 8) and individual interviews (n = 23). One diabetes nurse specialist took part both in a focus group and a subsequent interview. Ten diabetes nurse specialists did not take part, due to sick or maternity leave (n = 4), lack of time (n = 3), or non-response (n = 3). Characteristics of participating diabetes nurse specialists (region and type) are shown in Table 1.

**Table 1:** Participant matrix (n = 30)*.

Region	Population**	Diabetes prevalence***	Community nurses n = 19	Hospital nurses n = 11

		%	N (% sampled) (% region)	N (% sampled) (% region)

**South (n = 7)**	1,162,112	5.0	5 (26) (83)	2 (18) (10)
**West (n = 9)**	1,083,011	5.2	5 (26) (71)	4 (36) (22)
**Dublin North East (n = 6)**	1,022,184	4.5	4 (21) (80)	2 (18) (11)
**Dublin Mid-Leinster (n = 8)**	1,320,945	4.4	5 (26) (71)	3 (27) (19)

* 1 diabetes nurse specialist from a focus group also participated in an interview. ** 2011 population (Public Health Information System Data Table). *** Estimated prevalence; type 1 and type 2 combined [29].

### Data collection

Semi-structured focus groups and individual interviews were carried out with hospital and community diabetes nurse specialists across Ireland. Interviews and focus groups were conducted between December 2016 and February 2017. They took place in participants’ workplace (i.e. offices within hospitals or primary care centres) or in hotels when interviews were arranged to coincide with conferences or meetings. All interviews were conducted by a single researcher (FR) with a background in Public Health and Health Services Research and no experience of working within the health service. Participants knew the interviewer as an independent researcher conducting the study as part of her PhD training. The researcher made her position as a non-clinician clear to participants at the outset of interviews. Participants were invited by email and were provided with an information sheet explaining the study aims and methodology. Ethical approval to carry out the study was obtained from the Clinical Research Ethics Committee of the Cork Teaching Hospitals.

**Suppl. File 1** were developed based on the findings from the national survey and two pilot interviews with one community and one hospital-based diabetes nurse specialist. The topic guides included questions about the diabetes nurse specialist experience delivering care, governance, working with other professionals in the community and hospital, strengths and weaknesses of the current nurse specialist service, and, in the case of new diabetes nurse specialists, their approach to establishing their role. Hospital diabetes nurse specialists were also asked about the introduction of the new community diabetes nurse specialist role. Some interviews were conducted as part of a broader study on the implementation of the National Programme for Diabetes so some questions focused on particular aspects of that programme [30]. The topic guide was modified after an initial set of interviews to pursue emergent themes. For example, additional questions were included about the challenges of working between primary and secondary care, and how nurses worked with other professionals.

Prompts and probes were used throughout the interviews to encourage discussion. For example, ‘Why do you think that is?’; ‘How have you responded to that?’ ‘What does this look like?’. Signed informed consent was obtained before each interview. All interviews were audio-taped and transcribed in full. The average duration of individual interviews was 40 minutes, and 1.5 hours for focus groups.

### Data analysis

Open-coding of transcripts was carried out with a broad aim of understanding the experiences of diabetes nurse specialists in delivering care. Analysis was data-driven according to the approach described by Charmaz. This approach draws on some of the principles of grounded theory: developing categories and analytic codes from the data rather than pre-conceptualising these; coding with gerunds and trying to code actions or processes to stay closer to the data and; using In Vivo codes to preserve meaning. The latter are codes based on special or innovative terms used by participants [31]. While the study did not aim to generate a hypothesis or theory, it did seek to uncover an understanding of behaviours, which aligns with the purpose of grounded theory according to Noble and Michell [32]. Two transcripts (one community diabetes nurse specialist; one hospital diabetes nurse specialist) were read and open-coded by two other members of the research team (SMH, NMG), and the analysis approach and emerging themes were discussed. Subsequently, codes were organised and refined with a focus on diabetes nurse specialist actions or behaviours (how they acted to support integrated diabetes care), the factors that led them to act this way, and any consequences of those actions. This stage of coding was organised by looking for actions, causes and consequences within each transcript. In some cases, there was partial evidence (i.e. a cause and action, or action and consequence) within the one transcript. Actions were then grouped according to conceptual similarity, and concepts were discussed with the research team. **Suppl. Table 1** shows an example of the coding process. Memo writing was used throughout, particularly to establish conceptual links between the diabetes nurse specialist actions, the conditions or causes, and the outcomes of these [31]. Throughout the analysis the language and expressions of diabetes nurse specialist were maintained to preserve meaning and context. NVivo (Version 11) was used for data management. To assess the face validity of the synthesized themes, we presented the findings to a sub-group of community-based diabetes nurse specialists to check whether they accurately represented their views. The consolidated criteria for reporting qualitative research statement was used to inform reporting of the findings [33]. Anonymised participant quotations from community diabetes nurse specialists (CDNS) and hospital diabetes nurse specialists (HDNS) have been selected to illustrate qualitative findings.

## Findings

### Overview of themes

**Figure 2:**
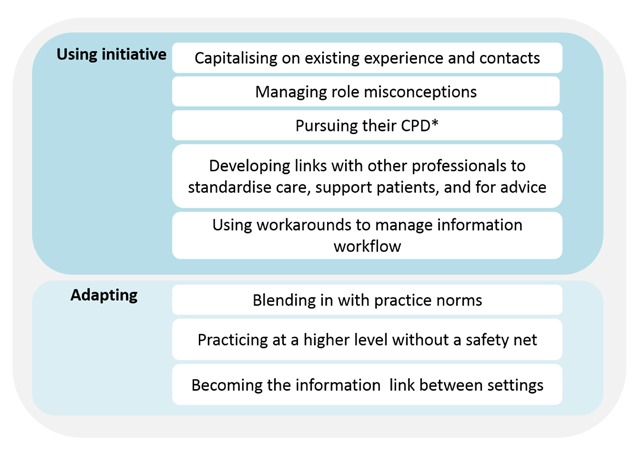
Examples of diabetes nurse specialists using initiative and adapting their role. * CPD, continuing professional development.

The overarching theme was encapsulated by the phrase ‘sink or swim’ which was used by community diabetes nurse specialists when describing their attitude to implementing their role. Community diabetes nurse specialists made a decision to ‘swim’, comprising two main behaviours (sub-themes); using initiative and adapting their role to the health service context (Figure 2). There were multiple examples of these two behaviours. Examples of using their initiative included: capitalising on their existing experience and contacts; managing misconceptions about their role; pursuing their own continuing professional development; developing links with other professionals and; using workarounds. Examples of adapting included: blending in with practice norms and needs; responding to the lack of a usual ‘safety net’ of the hospital-based multidisciplinary team, and; becoming “the only [information] link” between primary and secondary care. Most the examples were specific to the community diabetes nurse specialist experience. Therefore, we present these examples as they relate to community diabetes nurse specialist, and where appropriate highlight similarities or differences with the hospital diabetes nurse specialist experience.

### Capitalising on existing experience and contacts

When community DNS were first employed, there was no clear governance structure in place to oversee their role. When the new positions were introduced it was the intention that an official Model of Integrated Care document would be published. This document would outline care delivery for people with type 2 diabetes and specify the roles of nurse specialists, practice nurses, and GPs. Community diabetes nurse specialists would provide specialist support in the community in the management of more complex patients, provide education and support for GPs and practice nurses, and deliver clinics in general practice, independently, or in some cases initially jointly with the practice nurse or GP. As this document was not published, there was no formal, agreed guidance on what the role should involve or achieve.

When first employed, diabetes nurse specialists who were not linked to an existing diabetes programme felt there was no one to oversee their role or provide logistical support. Community diabetes nurse specialists’ overarching attitude was described as “sink or swim” (CDNS5, CDNS24) when establishing their role locally. To establish their role there was an onus on them to work with the situation they found themselves in, that is, ‘sinking’ was not considered a viable option. To ‘swim’ they had to draw on their own resources and skills, selling their role and service to local GPs and practice nurses to enrol practices:

So, at the moment I still think we are doing a bit of a sales job on our own surface [turf], we are going out selling what we do. So, if people hear about us and they want us to see somebody, usually again what happens is they either ring us or they email in to us and we’ll go out and meet them (CDNS14).

To reach GPs, they also used existing contacts or knowledge they had from previous positions or took advantage of visits made by pharmaceutical reps to practices, study days or information events. In areas where the role was entirely new, nurses had to “start from scratch” (CDNS1, CDNS5) in some cases generating contacts with GPs through cold calls:

One [practice] rang and asked me to come for a meeting which I did and started a clinic there. And no contact from anybody else. Had to start going around and making calls, and then, knocking on doors. (CDNS25).

So it’s a case of using my contacts that I previously had. It was hard at the start [laughs] but only because I had experience in [hospital] … there was nobody else to say right this is the way you should do it, because nobody else had a clue? (CDNS5).

### Managing role misconceptions

When community diabetes nurse specialists were first introduced, there was a lack of clarity about their role among other staff, and they had to manage misconceptions by 1) using initiative to clarify and explain the role and 2) asserting their role boundaries. Some hospital diabetes nurse specialists saw the community role as a different role to their own, while other hospital diabetes nurse specialists saw it as part of the hospital team “complementary to” (HDNS13) or a version of their own role:

It’s a valuable service I think really and can help to keep people out [of hospital], but in terms of what it helps to secondary care I’m not sure really. It’s more of us, it’s an extension of what we were doing (HDNS4).

Where community diabetes nurse specialists were perceived as separate to the hospital team, it was difficult to integrate care:

I think it would have been much more helpful if the consultants and the hospital-based team were engaged, were aware of what the role was, and that you were part of that team… The idea is that we’re meant to integrate care, but you can’t integrate any kind of care, you can’t integrate anything if your team aren’t on board. (CDNS7)

Community diabetes nurse specialists described how they managed misconceptions: by explaining their role, educating other staff, and establishing role boundaries, or justifying the need for flexibility in their role to managers (i.e. their working hours, how they spent their time, and tasks performed). Where community diabetes nurse specialists had faced a lack of understanding from managers, managing misconceptions sometimes involved organising their own hours, forgoing explanation to save time:

People are going to wonder what is your role, or what you can and cannot do, or maybe a public health nurse thinks that you can go in and give insulin every day, or…So, I think you just would need quite good interpersonal skills, and explain, ‘Well, no, that’s not part of my role, or…’ (CDNS#19).

Although in a much more established role, hospital diabetes nurse specialists had faced a similar scenario when their role had first been introduced. They also felt their role had not been appreciated or well understood. Other staff did not use the role appropriately, sometimes referring patients to them unnecessarily. Managing these misconceptions through ongoing education by hospital diabetes nurse specialists, together with an increasing number in place, meant that understanding of the hospital-based role developed over time. There was an expectation that understanding of the newer community diabetes nurse specialist role would develop in the same way:

Maybe about 5 or 6 years ago, we were getting a phone call just because they had diabetes. It didn’t matter really, they just saw ‘diabetes’ and they’d asked us, from the nurses on the wards, or from the doctors. But I think they’re appropriate referrals now and they tend to know when to call us. they probably realise that… We’re trying better. We’ve done a lot of guidelines, and a lot of input on how to manage somebody with diabetes when they come in for procedures. (HDNS23)

### Practicing at a higher level without a ‘safety net’

Community diabetes nurse specialists had to adapt to “a whole different MDT [multidisciplinary team]” in the community, and work without the “safety net” (CDNS14) usually present in the hospital, that is, equipment and supplies “on tap” (CDNS10), and other experts to check with who act as “backup” (CDNS22) for one another:

I’ll get in my car and I’ll drive off. You perhaps haven’t got the people around to bounce ideas off. You’ve got to be the one making some decisions. But also as well for your own planning and stuff, nobody comes to me and says, ‘Oh, there’s your clinics.’ You are responsible for your own workload…So it is a different role, you don’t have as much as a safety net of the team that you would do in a hospital, you are very much more… in some ways you can be more isolated but I prefer autonomous to isolated (CDNS14).

They adapted to the lack of this traditional ‘safety net’ by “practicing at a higher level” (CDNS22). Practicing at a higher level, meant asserting themselves as autonomous practitioners, and assuming greater responsibility and ownership over their workload and role organisation, for example, locating services in the community to refer to and link in with. It also involved exercising greater autonomy in clinical decision-making as they were now relied on as the “diabetes expert” (CDNS22) in GP practices, a “daunting” (CDNS22, CDNS11) prospect for some. To support themselves in this latter role they required confidence in their abilities and needed to maintain their skills and have a “much broader knowledge” (CDNS7) to deal with the patient mix and range of recommended medications:

You are expected to make decisions and to be advising the GP I suppose technically on paper, but I mean the GP is looking to you as a diabetes person to give the best advice on what we should do with particular patients. So, you are practising really at a higher level in primary care than you are within the hospital. (CDNS22).

The dynamics [in community] are different. I wouldn’t have been aware of the way things are done in primary care. It’s very different to the hospital. You have everything at hand in the hospital really. It’s very different out in the community. You have to look for services. You have to see what’s available. It probably took me a good 12 months settling in period. That’s just to get to know the system. (CDNS21)

Both community and hospital diabetes nurse specialists recognised the need to further their specialist skills, however owing to the lack of protected time and resources they had to use their initiative to participate in their continuing professional development on their “own time” (HDNS8). As a result, undertaking some professional education was considered unfeasible, for example, becoming a nurse prescriber. This course required an extended period of study leave, with a lack of remuneration for a “very big responsibility” (HDNS3):

I feel you have to be more up to date with all the medications and doses and side effects…Because you’re advising the GP what to do, at the end of the day, whereas you would have always had somebody to run that off. But then, I’m in it now [omitted] years, and I probably feel more *au fait* and on top of my drugs, [laughing] than I did before. …. I think you have to be quite confident in your own practice, but if you are, then it’s fine (CDNS19).

### Developing links with other professionals

Both community and hospital diabetes nurse specialists used their initiative to reach out to other professionals, for support and guidance, to share information and standardise care, or to support patients.

#### Creating an alternative safety net

While pursuing their continuing professional development was one way to support themselves to practice at a higher level, community diabetes nurse specialists also developed links with other professionals to create an alternative ‘safety net’. They created this ‘safety net’ by: 1) linking in with other community diabetes nurse specialists for advice, to be “shown the ropes” (CDNS21), to discuss concerns about patients, to compare service delivery with colleagues and, to learn from diabetes nurse specialists who were in their post longer and; 2) linking in with hospital colleagues for advice and to up-skill through case discussion. For some community diabetes nurse specialists, the 80/20 Whole Time Equivalent (WTE) split between time spent in the community and hospital settings had not yet been established. This delay left diabetes nurse specialists feeling “isolated” (CDNS16).

#### Developing links to standardise care

Community diabetes nurse specialists, faced with a lack of guidance on their role and a usual ‘safety net’ of resources and other colleagues, focused on aligning their role with that their peers. Community diabetes nurse specialists spoke about developing links to compare their role to other diabetes nurse specialists, and to ensure they were delivering their role correctly, or at least in a similar way to their peers. In this sense, they were standardising care, however it was different from the situation with the hospital-based role. Only hospital diabetes nurse specialists spoke about standardisation specifically in terms of guidance. Hospital nurse specialists linked in with peers to respond to the lack of national guidance on diabetes management, which meant guidance was developed at individual hospitals. Some hospital diabetes nurse specialists could reach out to other hospital diabetes nurse specialists to develop standard guidelines, harnessing existing nurse networks, to avoid “all reinventing different ones [guidelines]” (HDNS4) or “starting from scratch” (HDNS17).

#### Developing links to support patients

Both community and hospital diabetes nurse specialists linked with public health nurses to identify and support patients who needed their service, that is, those who do not attend a GP or hospital services and “can fall through the gaps” (CDNS26). Community diabetes nurse specialists used their initiative to expand the range of professionals they interacted with, liaising with public health nurses, and not restricting their contact to primary care professionals and the diabetes team in secondary care (CDNS5). Both community and hospital diabetes nurse specialists benefited from the knowledge and links public health nurses had, but also supported public health nurses in their role, through provision of education and advice (**Suppl. File 2**):

I know we link in with the GP, ultimately but you have to think of the bigger picture. Fair enough you have to say grand you don’t refer to me, I don’t accept referrals through the PHN but I can listen to what she has to say and I can get her to link in with the GP and get the patient sorted instead of saying I don’t have anything to do with them (CDNS5).

### Blending in with practice norms and responding to practice needs

In contrast with the autonomy they had in establishing their role, community diabetes nurse specialists relied on GPs to facilitate their role in general practice, as they “couldn’t go in solo and do our own thing” (CDNS24). Although confident in their own abilities, community diabetes nurse specialists were an unfamiliar professional when they first started in a practice. To build GPs’ trust in their role, community diabetes nurse specialists needed to adapt and “blend in” (CDNS#27) with how things were done in the practices and to be flexible and responsive to practice needs. This response was based on necessity and pragmatism:

You can’t be too dogmatic. You barely get in the door of a practice so you can’t be dictating everything to them, you know. You’re not going to muddy the waters. It takes a long time to build up trust with a GP practice so they’ve to trust you, you’re a complete stranger walking in the door to them, they don’t know you from Adam. (CDNS#10)

Blending in was achieved in different ways: taking steps to build trust with GPs; modifying their role to meet practice needs, and; fitting in with practice workflow. They built trust by respecting the GP’s autonomy, for example, remembering to “run everything by them” (CDNS10), and including GPs in medication decisions where feasible. Part of ‘blending in’ sometimes meant deciding not to pursue nurse prescribing. Community diabetes nurse specialists’ involvement in nurse prescribing depended on their situation with respect to the practice, that is, whether they were starting a new service or joining an existing primary care initiative. If community diabetes nurse specialists felt they were “hardly inside the door” (CDNS10) rather than in an area where they had “already built that trust and relationship” (CDNS22), they saw nurse prescribing as a challenge to GP autonomy which would remove opportunities for relationship-building, and they did not pursue it.

To blend in, nurses modified how they delivered their role in practices; community diabetes nurse specialists were flexible about the referrals they accepted, recognising that patients referred to their role differed according to practice confidence and experience in diabetes:

So, I ask them to send the newly diagnosed patients to me so that varies from practice to practice because some practices are maybe doing diabetes 20 years and some are new to it. So, then the ones that are new to it mightn’t have a practice nurse so they send everything to me, and then ones who are doing it a while would send the complex type news to me. (CDNS5)

This flexibility, these efforts to ‘blend in’ were also borne out of a recognition that practices differed in their experience levels. They needed to marry their goal of treating complex patients with their role as educator, responsible for building expertise and capacity in the community. In line with the original plan for the nurse specialist role, they supported GPs to develop their skills and expertise, for example ensuring GPs were informed of, and understood, any treatment changes. To do they often had to be responsive to practice workflow, creating time to discuss their decisions with GPs, waiting until the “doctor has the headspace” (CDNS16) or developing workarounds in cases where they did not have access to the GP:

I just can’t emphasise enough how flexible you have to be when you’re working in the community, and you have to acknowledge that you’re going in to somebody’s private business and that, it’s very much defined by the personalities in it. And it’s not all, the GP, it could be the nurse, you know. But you have to blend in with how things are done (CDNS#27).

The type of service community diabetes nurse specialists provide to practices, including the patients they see, is something which can change over time, as experience at the practice builds.

“I had to call out to them [the practice] a few times and show them how to set up a practice, show them how to educate patients, how to use the meter, show them what literature to use, start from scratch and now he’s [the GP] fine. They see the newly diagnosed, uncomplex, and now they send the complex to me.” (CDNS5)

### Using workarounds to manage information flow

Workarounds were an example of both sub-themes, ‘using initiative’ and ‘adaptation’. More specifically the *decision* to use a workaround was an example of initiative, while the *nature* of the workaround illustrated adaptation and modification of the role.

Community diabetes nurse specialists provided information to secondary care to inform management decisions. However, patient follow-up after community diabetes nurse specialists left GP practices, case discussion with consultants, and fast-track of patients to specialist services, were hindered by a number of factors. These factors were the absence of a shared record between settings, and GP ownership over patient data with no standard for how diabetes nurse specialists could safely share or transfer information out of the practice. As a result, diabetes nurse specialists were not always aware of what had taken place during a patient’s hospital or GP appointment. Operating between primary and secondary care, community diabetes nurse specialists used their initiative to develop workarounds which could address these information gaps.

### Becoming the information link between settings

The nature of these workarounds meant adapting to a complex information environment, becoming “the only link [or] bit of integration between the hospital and GP” (CDNS#24). They adapted by bringing back “basic” data (CDNS14) to the hospital and entering that, or filling out information twice, once in practice, and again on the hospital system, a “time-consuming” (CDNS21) and “frustrating” (CDNS15) process, checking patient information, phoning the hospital or e-mailing colleagues. Others used their initiative to manage the information deficit, for example, establishing a patient passport or their own system to remember patients, using the clinic dates and patient visit order. They recognised the risks inherent in these approaches, in relying on memory and notes. Sometimes, filling in information gaps meant unnecessary appointments in secondary care could be avoided.

These approaches contrasted with situations where community diabetes nurse specialists were based in a primary care centre, arranged for referrals to be sent directly to them, and established their own system for recording patient data electronically which gave them ownership over that data.

“You have the issue of patient information belongs to the GP. But I might have to ring a particular person about their insulin, but I’m not supposed to have that information beside me. So if I have 20 people to ring, how am I supposed to remember exactly all of those people, and be safe in doing that?” (CDNS7)

## Discussion

This study found that the capacity of diabetes nurse specialists to adapt and innovate is important for implementation of their role in real-world frontline practice. For community diabetes nurse specialists in particular it enabled them to work with, and around, features of the outer and inner setting. Aspects of the inner and outer setting which are important for implementation have been synthesised in the CFIR [14]. Some of these aspects overlap with determinants identified in the current study; inter-organisational networks and connectedness (i.e. general practice delivery by independent self-employed practitioners, absence of a shared electronic patient record between primary and secondary care), and intra-organisational culture and norms (i.e. a lack of role understanding by peers and managers, peer network support for autonomy and shared learning, and practice workflow, organisation and experience in diabetes management). These determinants of community diabetes nurse specialist behaviours highlight the realities of introducing boundary-spanning roles to facilitate integrated care when the wider system is not yet configured or prepared to support this model; the response being pragmatic efforts to optimise the role where feasible. In short, new nurse specialists represent a somewhat isolated strategy to integrate care. The nurse role, which does perform functions to integrate care, but is challenging to implement within a resource-constrained and misaligned infrastructure. The determinants of practice organisation and experience, absence of a shared electronic patient record, a lack of role understanding by peers and managers, and the nurse specialist response to them suggests certain strategies are required to move the role beyond one which is just ‘workable’ to one which also effectively integrates care delivery.

### Integrating information

In line with existing work, the current study highlights the importance of integrated data systems and data-sharing in the delivery of integrated care [15,17,18]. There is no shared electronic record between settings in Ireland, and inefficiencies in data-sharing and documentation and clinical information systems were identified. Poor coordination and information systems between secondary and primary care continues to pose a problem for integrated care [18,27,34]. The current study identified the specific consequences of this issue for implementation, namely, curtailing aspects of the role such as case discussion and follow-up; placing additional demands on time, including liaison to address information gaps and duplication of data entry; as well as missing opportunities to streamline services and appointment slots. While building the ICT infrastructure has been recommended in existing policies, including the recent Irish policy document Slaintecare [23,35], the study findings represent tangible examples of how un-linked information systems present a day-to-day problem when managing patients. Specifically, in Ireland, as part of the eHealth strategy interoperable EHRs have been piloted, with provisions for the operational use of the national Individual Health Identifier [36].

### Role ambiguity

The fact that both community and hospital diabetes nurse specialists shared experiences of role misconceptions indicates the persisting challenges of introducing new clinical roles, and the need for greater clarity on nurse specialist roles integral to integrated care to ensure they are used appropriately and effectively. Inter-professional relationships [15,16], and understanding of new roles [17] are important in the delivery of integrated care. Role ambiguity is an international challenge in the establishment of advanced nursing roles [37,38]. As evident in the current study, ambiguity can lead to inappropriate or ineffective use of the role [17], and hinder interdisciplinary collaboration [17]. With professionals like community nurse specialists increasingly working across care boundaries, issues around role understanding and acceptance, blurring of professional roles and responsibility may continue [39,40]. A systematic review of barriers to primary care type 2 diabetes management identified “uncertainty and unease” about clinical responsibility when coordination across numerous professionals occurred [41]. With regard to GP-led integrated care in Australia, there was a need to build trust and change the “mindset” of specialists to recognise the benefits of moving complex diabetes care to the community, and to counter resistance from GPs who did not want to “deal with” more complex management [42]. While both community and hospital diabetes nurse specialists in this study did manage role ambiguity, this is an issue which may be circumvented through advance planning, rather than placing expectations on clinicians to explain and justify their role. Future efforts to integrate care will need to consider how to generate receptiveness to new roles and new ways of interdisciplinary working. Strategies to prepare for this new role could include ensuring readiness in terms of infrastructure and resources [43], making policies and protocols which outline the role available [37,44], formally designating an individual (e.g. local nurse administrator) to oversee implementation and facilitate systems entry [43,44], and engaging stakeholders [43], in particular influential or senior professionals to ‘champion’ the role within the organisation [44,45]. Since this study was completed, the National Clinical Programme for Diabetes has developed a guidance document to help community diabetes nurse specialists to explain their role and introduce their role in new practices.

### Supporting greater autonomy

Internationally, nurse specialists have become more autonomous [25,46] and have moved to the community setting to facilitate the integration of care [7,24,47]. Existing research indicates that nurse specialists who work in this setting may face professional isolation [48]. This aligns with the finding from the current study that nurse specialists work without the usual ‘safety net’ of other experts and a link to the hospital. This distinction made between isolation and autonomy may depend on the accessibility of continuing professional education, the ability to foster links to secondary care professionals, and the availability of peer support, which created an alternative to the ‘safety net’ originally lost by moving to the community. Indeed, peer support [49,50,51] and engagement in communities of practices [17], have been identified as facilitators of the nurse specialist role [49,50].

In the current study, engagement in professional networks also provided an important avenue for peer learning, sharing knowledge and developing specific skills, (e.g. care coordination, working with different providers, promoting service engagement), which cannot be supported through formal training. Both community and hospital diabetes nurse specialists in our study reported limited study leave, however, time to engage in education and capacity-strengthening is not an issue unique to Ireland [17,46]. Working in boundary-spanning roles requires a blend of ‘formal and tacit knowledge’ [52]. Adequate training for boundary-spanning roles created to support the integration of care [52] is increasingly important to ensure these roles are sustainable, do not rely wholly on ‘exceptional’ and committed individuals with local links [52], and can be replicated in the event of staff turnover. In Ireland, there is a commitment to building the advanced nursing infrastructure to support chronic disease management in the community [53], with the Slaintecare report outlining a plan for new Advanced Nurse Practitioner demonstrator posts [23,35]. Peer networks can act as a ‘safety net’, providing nurses with confidence in their clinical decision-making when working in the community, and facilitating shared learning among increasingly autonomous community-based professionals (including advanced nurses). Given this, building and supporting access to peer networks should form a central part of efforts to integrate care both in Ireland and internationally.

### Education and training

Training should extend beyond boundary-spanning roles and encompass system-level changes in the education of *all* professionals involved in the integrated care. This should include better support for further training in diabetes in general practice which would complement the nurse specialist role in upskilling and building team capacity. Training in new ways of working may need to begin at the undergraduate and postgraduate level and be supported by on-going learning and continuing education. This may address the specialisation and “siloed nature of training” [52] of healthcare professionals, which can challenge collaborative working across professions and settings.

The study findings illustrate how community diabetes nurse specialists offer a valuable contribution to building and developing the primary care team capability, through their role as clinical expert providing peer support. However, their success evidently depends on their ability to adapt and fit in with practice needs and workflow. While interventions may be made ‘workable’ at a local level, their implementation will also be affected by the system capacity (social-structural resources available to those enacting implementation) and whether it enables professionals like community diabetes nurse specialists to contribute to the implementation process [54]. While nurse specialists may aim to build practice team capacity and skills, the time needed to engage with GPs and practice nurses to achieve structured development and education may not always be available. This was evident in the workarounds used by nurse specialists to communicate with busy GPs. Practice education may need to be specifically resourced to facilitate effective engagement, for example, incorporating time for nurse specialists to work with practices to systematically identify their educational needs, and develop explicit practice plans [4].

### Capacity for flexibility and innovation

Embedding change and routinising multidimensional social interventions successfully into complex, adaptive health systems is a challenge [55]. Implementation will be affected by the degree to which the intervention is workable in, and can be integrated into, existing practice [54]. Our findings illustrate the ongoing contribution of nurse specialists to embed change by cultivating trust and building relationships with GPs and managing role misconceptions among peers and managers. When implementing and evaluating new models of integrated care, as asserted by Foster et al. in their study of GP-led integrated care [42], there is a need to “balance the ‘ideal’ model with the realities of resourcing”. It is important to allow for interventions to be adapted to the local context, rather than insisting on rigid standardisation [56]. Our findings illustrate the creative, self-organising behaviours [57] inherent in complex systems, and how flexibility and capacity for innovation may be important in professionals leading and implementing integrated diabetes care. When implanting professionals in new roles such as these, attention should be given to these skills which will support ‘workability’ in a range of different contexts.

### Clarity on the core elements

Our findings also illustrate how, in seeking to make integrated care ‘workable’, providers make trade-offs between achieving intervention fidelity and sustaining care delivery. The adaptations made by community diabetes nurse specialists to their role and the model of integrated care to make it ‘workable’ can be classified as intervention content modifications [58]: 1) adding elements consistent with the principles of integrated model (e.g. reaching out to, and educating public health nurses); 2) refining the intervention to make it more appropriate (e.g. being flexible in referrals, using workarounds to fit in with practice workflow and manage information gaps); 3) removing elements (e.g. nurse prescribing). However, this raises the question about which elements of community diabetes nurse specialists role to support integration are ‘core’ and which belong to the ‘adaptable periphery’ [14]. Some variation is to be expected in complex systems; removing it entirely may limit possibilities for innovation and creativity [13]. In Ireland, diabetes management in general practice ranges from ad hoc and opportunistic to structured approaches, some of which are driven by formal primary care diabetes programmes [20,21]. We might expect variation in the role delivered by community diabetes nurse specialists to GP practices depending on GP experience and the nature of the GP-diabetes nurse specialist relationship. The community role is still in its infancy and some elements may be accorded some flexibility in the earlier stages of implementation. As the role develops however it will be important to support community diabetes nurse specialists to navigate the “dance between flexibility and consistency” [59], providing some specification, and clarity around which elements can continue to be adapted, and, if deemed essential, how these can be consistently implemented [13]. Some guidance is needed on how to suitably modify nurse specialist service delivery, if required. In Ireland and internationally, the focus is often on developing interventions that work, but less so on how to guide delivery of services and interventions once already in place, or how to adapt them so they are still effective [60]. Continuing to monitor and adapt interventions *during* delivery can identify important influences on service delivery which may not have been prioritised, were missed, or simply not apparent before implementation began [61]. Integrated care should continue to be evaluated as it is rolled out to determine what adaptations occur, why and whether they influence effectiveness. This may be particularly relevant in the Irish context as the Department of Health move towards developing key performance indicators for integrated nursing roles as part of demonstrator projects [62].

## Strengths and limitations

We believe findings from our study are transferable to other countries facing similar health service constraints e.g. poor integration across service providers [27] lack of shared record or incompatible information systems between primary and secondary care [18,63], GPs working as independent practitioners. Moreover, the clinical responsibilities and core competencies of diabetes nurse specialists are similar internationally [25,46,64]. That the researcher who conducted the interviews was not a clinician may be a limitation; some authors suggest when interviewing clinicians, peer researchers can enlist greater trust and may be able to elicit richer data on more sensitive topics [65]. However, being a non-clinician ‘outsider’ also meant the researcher had no preconceptions or opinions about how the nurse specialist role works in practice and may have been less susceptible to what Chew-Graham and colleagues refer to as “shared conceptual blindness” [65]. Almost all community-based diabetes nurse specialists were sampled for this study. However, since a lower proportion of hospital diabetes nurse specialists were invited to take part their perspectives may not be as well-represented. While coding actions or processes allowed themes to be guided by diabetes nurse specialist responses, in line with the data-driven principle of grounded theory, core behaviours only became apparent during the later stages of analysis. The study was not designed to specifically explore how interventions (including the diabetes nurse specialist role) are adapted; a more nuanced understanding of the process of adaptation may have been achieved had this been the sole aim. An established conceptual framework such as the CFIR was not used as a guide during the analysis. Had the aim been to elucidate diabetes nurse specialists’ views on specific determinants, using CFIR to structure the topic guide may have been beneficial. This may have prompted a discussion around other elements of the outer context, for example, financing and incentives, leading the researcher to probe whether and how this influenced the nurse specialist role. As it stands, that these factors were not discussed suggests their impact may be less apparent or important to nurse specialist when reflecting on their service delivery, as compared to other factors such as peer relationships. A final strength is the fact that when we presented the findings to a sub-group of community-based diabetes nurse specialists they expressed recognition of the behaviours identified.

## Conclusion

Our findings have implications for the implementation of integrated care internationally. Successful implementation and spread of integrated care models supported by nurse specialists requires a combination of strategies to address determinants in the outer and inner context. The findings highlight the important preparatory work and relationship building that are key to successful role implementation. Interoperable EHRs between settings are required to support nurse specialist to work across care boundaries. Strategies to avoid ambiguity when introducing new roles to support integrated care are important to ensure their appropriate and effective use; this includes engagement and consultation with managers prior to through the introduction of new roles. To support greater autonomy specialist nurses should be facilitated to engage in education and training, and to link in with peer networks and other professionals. Further training and resourcing at a practice level may be necessary to facilitate specialist nurses in their role building practice team capacity. An ability to adapt, and a capacity for flexibility and innovation, can facilitate the implementation of integrated care delivery into existing practice and specific contexts, and these attributes should be considered when introducing new boundary-spanning roles. However, there is also a need for clarity and guidance on core elements, to support standardisation of new roles and new care models. Integrated care should continue to be evaluated as it is rolled out to determine what adaptations occur, why and whether they influence effectiveness.

## Additional Files

The additional files for this article can be found as follows:

10.5334/ijic.4215.s1 Suppl. File 1. Topic guides.

10.5334/ijic.4215.s2 Suppl. File 2. DNS behaviours in relation to Public Health Nurses (PHN) which facilitate delivery of the DNS service and support PHNs in their role.

10.5334/ijic.4215.s3 Suppl. File 3. What this study adds.
