# Supplementary material for: ‘Sink or Swim’: A Qualitative Study to Understand How and Why Nurses Adapt to Support the Implementation of Integrated Diabetes Care
Source: Int J Integr Care. 2019 Apr 3;19(2):2. doi: 10.5334/ijic.4215 (PMC6450245; doi:10.5334/ijic.4215)
Supplement: Suppl. File 1. — Topic guides. [file ijic-19-2-4215-s1.pdf]

Topic Guides

Topic guides exclude sections which focused specifically to other aspects of the National Programme for Diabetes (i.e. RetinaScreen, Model of Care for the Diabetic Foot) being explored as part of the broader study.

National Clinical Programme for Diabetes
**Rationale:**<br>The role of the Diabetes Nurse Specialist is central to diabetes care and continues to evolve in response to the policies and practices within the health system. The number of Diabetes Nurse Specialists working in Ireland has increased in recent times, due in part to the changes introduced by the National Clinical Programme in Diabetes (NCPD). We want to know what you think about these changes, and how the national programme has impacted on your local service. As you know, we recently conducted a national survey of Diabetes Nurse Specialists in Ireland to assess the availability of specialist services in Ireland. We now want to hear about your experience providing diabetes care to understand in more detail, the challenges and opportunities for integrated care.<br><br>We did some preliminary interviews about how services implemented as part of the NCPD are working so I would like to ask you about some of the theories that have come up and what your experience has been

The interview should last about 30 minutes

Just some general house-keeping before we start (Briefly go through consent form)

- If it is ok with you I will audio record the interview so I can give you my full attention and don't have to take any notes. This way I can be sure I don't miss anything.
- Anything we discuss will be confidential and your identity will remain anonymous on any reports or publications. We may use direct quotes from this interview but again I stress that your name will **not** appear anywhere. Your identity and position will be kept completely anonymous.
- Finally you can stop the interview at any point, if you wish. And you are free to withdraw from the study at any time.
- Do you have any questions for me before we get started?
- Sign consent and give copy.

NCPD outline

The National Clinical Care Programme for Diabetes was set up a couple of years ago. It brought together representatives from all the different disciplines involved in diabetes care to try and improve the way services are delivered. They do this in a number of different ways, for example designing models of care for patients or trying to secure additional resources and posts for diabetes. The National Programme was instrumental in developing the new model of integrated care (including the recruitment of integrated care nurses), the national retinopathy screening programme, RetinaScreen, and developing a standard Model of Care for the Diabetic Foot (including the recruitment of additional podiatrists etc).

TOPIC GUIDE (Community DNS)	
**<u>Service provision/ DNS role</u>**	
Can you tell me a bit about the diabetes service you provide here in XX? (i.e. Type of patients, referrals, where are you based, how does governance work)	
What is your role in the community?	Has this changed over time? How?<br>What is your role for the 1 day in the hospital?
How did you set up the service in this area?	What was your approach to contacting GPs (day 1)? List of GPs?<br>What was your approach to GPs who may not link in?<br>What happens with those who do not engage with the service?<br>Are all GPs able to access the service? Why (not)?
**<u>Working with other professionals / across settings</u>**	
How has your service received in your area?<br>By GPs, practice nurses, In the hospital by consultants, by other DNS, patients?	Why do you think this is?<br>How have you responded?
How has your role been received in secondary care?	
Do you have a liaison role with other professionals in the hospital/community?	With who? What does this involve/look like?<br><br>Is there an agreement for how your service should work (primary & secondary care? What does this cover? How does it work?
Anything which could be done differently in terms of the DNS role to facilitate working with other services/professionals?	Between primary & secondary; within secondary care or the community?
How do you find being based in both primary and secondary care?	What are the challenges?
**<u>Service changes</u>**	
Have you seen any recent changes in how patients are managed between primary & secondary care here?	What was this change? What do you think the impact of this is on patient care? On your own work?
Would you describe care as integrated[1]? (Why/Why not?)	

[1] What we mean by integrated care is that patients are managed by primary and secondary care services depending on the complexity of their diabetes. There are good links between primary and secondary care (e.g. better access to hospital services for integrated care patients) and professionals in both sectors have an

Do you follow the national model of care[2] in this area?<br><br>Are you familiar with the National Clinical Programme for Diabetes?	
**Integrated Care**	
**GP engagement with DNS**<br>GP engagement with the new integrated DNS service has been varied (by which we mean in some areas DNS couldn't 'get in the door' in other areas they were 'welcomed').<br><br>Would this fit with your experience?	Why do you think that is?
**Role of DNS**<br>It has been suggested in previous interviews that there is variation in the nurse role in different areas. So the nurses have different roles[3] in practices (e.g. see different patient types of patients)<br><br>What does your role involve in the practices you work with?<br><br>Would you say your role varies - are there any parts you feel don't happen in certain places?	Why do you think that is?
**Integrated care in secondary care**<br><br>We are trying to find out how the integrated role and the model of care are being implemented in secondary care.<br><br>What has your experience been?	Is it clear how the model of care is meant to work in secondary care?<br>(e.g. discharge back to community)<br><br>Anything you think needs to be done in secondary care services for the model of care to work? (e.g. standardisation of GP referrals forms to facilitate discharge; discharge + advice to GPs)<br><br>-Has the model of care been seen as a positive or negative change? If so, why?

understanding of where different patients should be cared for. So this would mean joint management of more complex patients, with less complex type 2 patients mainly managed in primary care.

[2] The national model of care aims to standardise management of patients with diabetes, including management across primary and secondary care. It outlines the different roles of those involved in care i.e. GP, practice nurses, DNS, dieticians, their roles and responsibilities, along with the types of patients to be cared for across the two sectors, and those to be cared in secondary care

[3] The intended role of the 'integrated' DNS was that they would act as a link between primary & secondary care, run clinics in primary care, provide training and support to practice nurses, serve as specialist support for GPs/ practice nurses for complex patients & support GP/ practice nurse in management of uncomplicated type 2 diabetes, be involved in structured education

	- Is it clear how your role is meant to work in the hospital? How does governance work?

Final questions	
What parts of the DNS role work well and what don't?<br><br>What facilitates or impedes you in delivering your role?<br><br>Is there anything that I haven't touched on that you think is important?	Any way the role could be changed or improved?

TOPIC GUIDE (Hospital DNS)	
**<u>Service provision/ DNS role</u>**<br>Can you tell me a bit about the diabetes service you provide here in XX? (i.e. Type of patients, referrals, where are you based, how does governance work)<br><br>**<u>Working with other professionals / across settings</u>**<br>How is your service received in your area?<br>By GPs, practice nurses, In the hospital by consultants, by other DNS, patients?	Has this changed over time? How?<br><br>Why do you think this is?<br>How have you responded?
How are patients managed between primary & secondary care here?<br><br>Would you describe care as integrated[4]? (Why/Why not?)<br>Do you follow the national model of care[5] in this area?	-Always been the case?<br><br>-Any change in how they are managed? (Why (not)?<br><br>-If change in secondary care….What was this change? Why? Impact of this is on patient care? On your own work?

[4] What we mean by integrated care is that patients are managed by primary and secondary care services depending on the complexity of their diabetes. There are good links between primary and secondary care (e.g. better access to hospital services for integrated care patients) and professionals in both sectors have an understanding of where different patients should be cared for. So this would mean joint management of more complex patients, with less complex type 2s mainly managed in primary care.

Are you familiar with the National Clinical Programme for Diabetes?	
**Integrated care**	
**<u>GP engagement with DNS</u>**<br>GP engagement with the new integrated DNS service has been varied (by which we mean in some areas DNS couldn't 'get in the door' in other areas they were 'welcomed').<br><br>Would this fit with your experience? Why do you think that is?	Why do you think that is?
**<u>Integrated care in secondary care</u>**<br><br>We are trying to find out how the integrated role and the model of care have been implemented in secondary care.<br><br>What has your experience been?	Clear how the model is meant to work in secondary care?<br><br>- Anything you think needs to be done in secondary care services for the model of care to work? (e.g. standardisation of GP referrals forms to facilitate discharge; discharge + advice to GPs)<br><br>-Has the model of care been seen as a positive or negative change? If so, why?<br><br>-How has the new 'integrated' DNS role been received in the hospital by consultants and other DNS? Seen positively or negatively? Why?<br><br>-Is it clear how this role is meant to work in the hospital? How does governance work?
**Final questions**	
What parts of the DNS role work well and what don't?<br><br>What facilitates or impedes you in delivering your role?<br>Is there anything that I haven't touched on that you think is important?	Any way the role could be changed or improved?<br><br>-At level of nurse (e.g. support networks; own experience)<br>-Wider infrastructure (e.g. space, staffing, ICT)

[5] The national model of care aims to standardise management of patients with diabetes, including management across primary and secondary care. It outlines the different roles of those involved in care i.e. GP, practice nurses, DNS, dieticians, their roles and responsibilities, along with the types of patients to be cared for across the two sectors, and those to be cared in secondary care
