# Supplementary material for: ‘Sink or Swim’: A Qualitative Study to Understand How and Why Nurses Adapt to Support the Implementation of Integrated Diabetes Care
Source: Int J Integr Care. 2019 Apr 3;19(2):2. doi: 10.5334/ijic.4215 (PMC6450245; doi:10.5334/ijic.4215)
Supplement: Suppl. File 2. — DNS behaviours in relation to Public Health Nurses (PHN) which facilitate delivery of the DNS service and support PHNs in their role. [file ijic-19-2-4215-s2.pdf]

Suppl. Box 1 DNS behaviours in relation to Public Health Nurses (PHNs) which facilitate delivery of the DNS service and support PHNs in their role

Identify patients

"You have to think beyond the box. Like I know we link in with the GP, like he ultimately but like you have to think of the bigger picture. Like fair enough you have to say Grand you don't refer to me, I don't accept referrals through the PHN but I can listen to what she has to say and I can get her to link in with the GP and get the patient sorted instead of saying I don't have anything to do with them" (CDNS5)

"They can fall through the gaps. It's all that kind of joined up stuff between community and hospitals and specialists and all of that, and then there's this little cohort of patients in community who sees nobody. They just...see the public health nurse, who is treating an ulcer, singularly" (CDNS3-FG1).

Contact PHNs to arrange for patients to receive insulin in the community

Liaise with PHN to follow-up discharged patients in community

Facilitate a faster turnaround for PHNs on prescribing or adjusting insulin

"They [PHNs] know that we adjust the insulin…then the other thing is that it's done that day, it's a time turnaround. It's fast. It's not waiting for a week or maybe calling the GP out to adjust insulin when it can be done from here" (HDNS17)

Facilitate or advise PHNs to link with the GP to get the "*patient sorted*" (CDNS5)

Facilitate PHN access to bloods or appropriate equipment

Provide PHNs with informal advice and education:

“*They [PHNs] were astounded to think that they would have to check a patient's glucose level before they would leave a dressing clinic, if they were a diabetic or on sulphonylureas. Because they didn't see it as being part of, a. the overall care and b. part of their role*” (CDNS7)

“*Because I'm in the open plan office they'll come by to run something by me. So they'll have learned a lot about diabetes”* (CDNS5)

Benefit from PHN knowledge:

“*They have direct links, they know the family dynamics and everything, they're on the ground”* (CDNS1)
