# Supplementary material for: ‘Sink or Swim’: A Qualitative Study to Understand How and Why Nurses Adapt to Support the Implementation of Integrated Diabetes Care
Source: Int J Integr Care. 2019 Apr 3;19(2):2. doi: 10.5334/ijic.4215 (PMC6450245; doi:10.5334/ijic.4215)
Supplement: Suppl. File 3. — What this study adds. [file ijic-19-2-4215-s3.pdf]

What this study adds

- This study highlights the realities of introducing boundary-spanning roles to facilitate integrated care when the wider system is not yet configured or prepared to support this model; the response being pragmatic efforts to optimise the service where feasible.

- The findings illustrate the ongoing contribution of community nurse specialists to embed change by cultivating trust and building relationships with GPs and managing role misconceptions among peers and managers.

- In the current study nurse specialists contribute to building and developing the primary care team capability, through their role as clinical expert providing peer support. Our results suggest their success is dependent on their ability to adapt and fit in with practice needs and workflow.

- Interventions such as nurse specialist-led integrated care may be made 'workable', through flexibility and using initiative. However, successful implementation of nurse specialist-led integrated care requires strategies to address elements in the inner context (such as differences in practice organisation, role ambiguity, peer network support for autonomy and opportunities for shared learning) and outer context (information systems)
